# Complete Genome Sequence of the Persistent Listeria monocytogenes Strain R479a

**DOI:** 10.1128/genomeA.00150-15

**Published:** 2015-03-19

**Authors:** Stephan Schmitz-Esser, Lone Gram, Martin Wagner

**Affiliations:** a Institute for Milk Hygiene, University of Veterinary Medicine Vienna, Vienna, Austria; b Department of Systems Biology, Technical University of Denmark, Kongens Lyngby, Denmark

## Abstract

The complete genome sequence of the persistent Listeria monocytogenes strain R479a isolated from smoked salmon in Denmark and belonging to lineage II, serovar 1/2a, and multilocus sequence type 8 (ST8) is presented here.

## GENOME ANNOUNCEMENT

The Gram-positive facultative intracellular pathogen Listeria monocytogenes is the causative agent of listeriosis, a rare but severe disease transmitted through the consumption of contaminated food (1). L. monocytogenes can survive and grow in a multitude of natural and man-made habitats (2). The long-term occurrence of genetically indistinguishable L. monocytogenes strains in the same food production plant over a long time period has been termed persistence. Some L. monocytogenes strains are able to persist for months or years in food production environments (2–4). Currently, two models are available to explain this persistence (2): either certain L. monocytogenes strains have unique phenotypic and genotypic characteristics facilitating long-term survival in food processing environments, or persistence is largely a random process and most L. monocytogenes strains can establish persistence if present in an appropriate niche at an appropriate time (2). Thus, persistent L. monocytogenes strains represent a big challenge for food safety; we therefore analyzed the genome sequence of the persistent L. monocytogenes isolate R479a (lineage II, serovar 1/2a, sequence type 8 [ST8]), which was isolated from smoked salmon from Denmark and persisted from November 1996 to January 1999 (5).

DNA was isolated using the Qiagen Genomic-tip columns and buffers, according to the recommendations of the manufacturer. Genome sequencing was performed using an Illumina GAII genome analyzer with paired-end sequencing technology and 100-bp read length, using Illumina standard protocols. A total of 3.057 million reads were used for a *de novo* assembly using Velvet, resulting in 33 contigs, with an average coverage of 140×. The remaining gaps were closed by PCR and Sanger sequencing (LGC Genomics, Berlin, Germany). Gene prediction and annotation were done using the MicroScope platform (https://www.genoscope.cns.fr/agc/microscope/home/ [6]). Multilocus sequence typing (MLST) was performed with the MLST tool available on the Center for Genomic Epidemiology website (https://cge.cbs.dtu.dk/services/MLST/ [7]).

The R479a genome consists of a single circular chromosome with a size of 2,944,998 bp and 2,995 predicted coding sequences, a G+C content of 37.9%, and a plasmid (pLMR479a) of 86,652 bp containing 92 predicted coding sequences and a G+C content of 37.0%. Interestingly, and in contrast to most other sequenced Listeria genomes, the R479a genome contains only 5 rRNA operons and 58 tRNA genes.

The L. monocytogenes R479a genome contains a typical Listeria pathogenicity island and a full-length internalin AB (*inlAB*) locus. R479a encodes 11 internalins and 17 internalin-like proteins. All virulence genes present in L. monocytogenes EGDe, except the homologues of *lmo0320* (*vip*) and *lmo2026*, are present in R479a.

pLMR479a is highly similar to other Listeria plasmids and carries many genes possibly involved in stress response or transporters for the export of heavy metals, such as a Tn*5422* copy. Interestingly, pLMR479a harbors a region of 14 genes (LMR479A_p0071 to LMR479A_p0083) of approximately 10 kbp, with a G+C content of 37.9%, in which all proteins show the highest similarity to proteins from *Firmicutes* other than Listeria species; this region had thus most likely recently been transferred into pLMR479a.

### Nucleotide sequence accession numbers.

The complete genome and plasmid genome sequences of strain R479a have been deposited in ENA/GenBank/DDBJ under the accession numbers HG813247 and HG813248, respectively. The versions described in this paper are the first versions.
